# Supplementary material for: Quorum sensing as a mechanism to harness the wisdom of the crowds
Source: Nat Commun. 2023 Jun 9;14:3415. doi: 10.1038/s41467-023-37950-7 (PMC10256802; doi:10.1038/s41467-023-37950-7)
Supplement: Supplementary file 1 — Supplementary Information [file 41467_2023_37950_MOESM1_ESM.pdf]

SUPPLEMENTARY INFORMATION

Quorum sensing as a mechanism to harness the wisdom of the crowds

Stefany Moreno-Gámez*, Michael E. Hochberg, G. Sander van Doorn

*Correspondence to: stefany@mit.edu.

Supplementary Figures

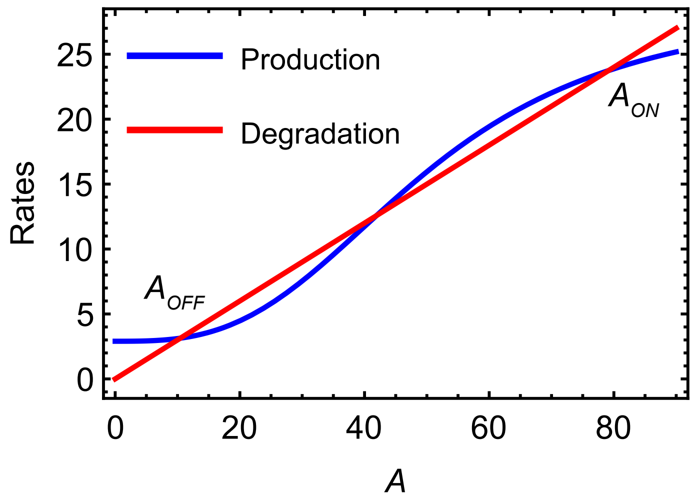

Figure S1: **Steady-states of the model for a single cell when $c = 0$.** In the absence of diffusion of A into the extracellular space, we chose a set of parameter values that renders the system described by Eq. (1) bistable with two stable equilibria, A_{OFF} and A_{ON}. The set of parameters is $n = 3$, $K = 50$, $d = 0.3$, $k_0 = 2.9$ and $k = 29$. Production of A occurs at a rate given by $\frac{k_0 + k\,(A/K)^n}{1+(A/K)^n}$ and degradation at a rate given by $d\,A$.

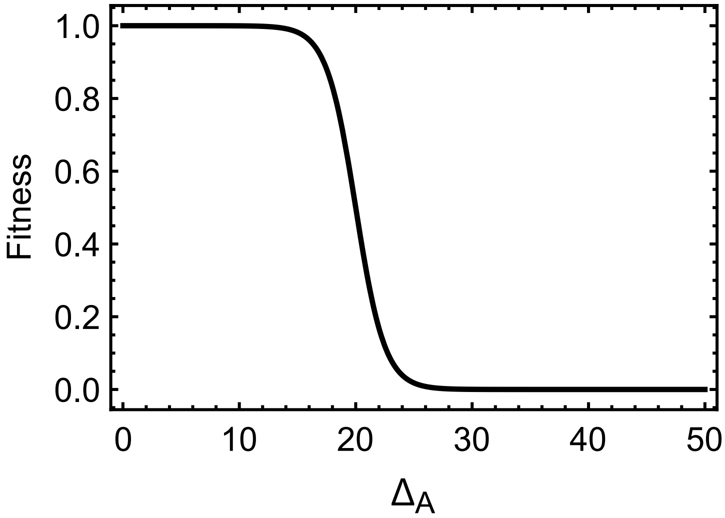

Figure S2: **Fitness function** $w(\Delta_A)$**.** Function applied at the end of one generation to determine the fitness of a cell depending on the difference Δ_A between its average value of A and the optimal expression level for the current environmental state.

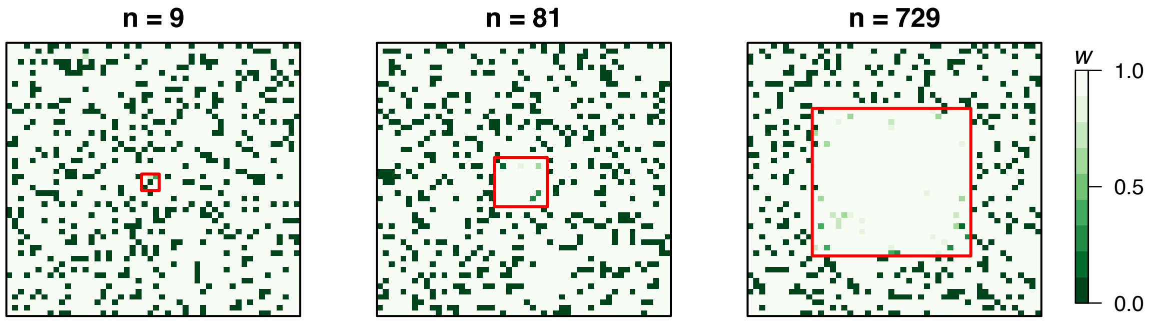

Figure S3: **Collective sensing becomes beneficial when cells are surrounded by other communicators.** Individual fitness values in three populations of non-communicators that contain a subpopulation of communicating cells located in the center of the 50x50 grid. In each panel, the size of this subpopulation is indicated by n and its location in the two-dimensional grid is delimited by a red square. For non-communicators, $c = 0$ whereas for communicators $c = 0.1$. Fitness increases from green to white and in each panel fitness values for every cell are calculated after simulating one generation where the population encounters an E_{ON} environment. A similar pattern is observed in an E_{OFF} environment. Other parameters are the same as in Fig. 2. Cells benefit from collective sensing only if they are surrounded by other communicators.

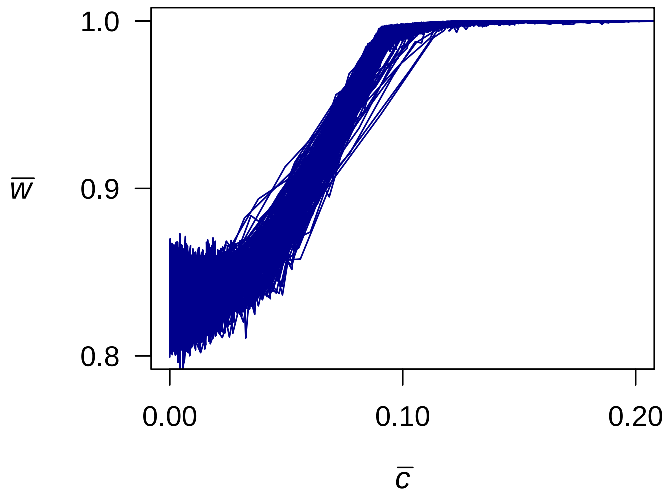

Figure S4: **Fitness increases marginally past a threshold value of $\bar{c}$.** Mean fitness as a function of the average c for the evolutionary trajectories presented in Fig. 2. Beyond $\bar{c} = 0.1$ the population is communicating to the extent that most individuals can correctly estimate the state of the environment every generation. Since the fitness function has a sigmoidal shape that strongly penalizes individuals for making a wrong estimate of the environmental state but not for small deviations from either A_{OFF} or A_{ON} (see Fig. S2), fitness increases marginally with $\bar{c}$ after a threshold value which corresponds to $\bar{c} = 0.1$ for the canonical parameters of our study.

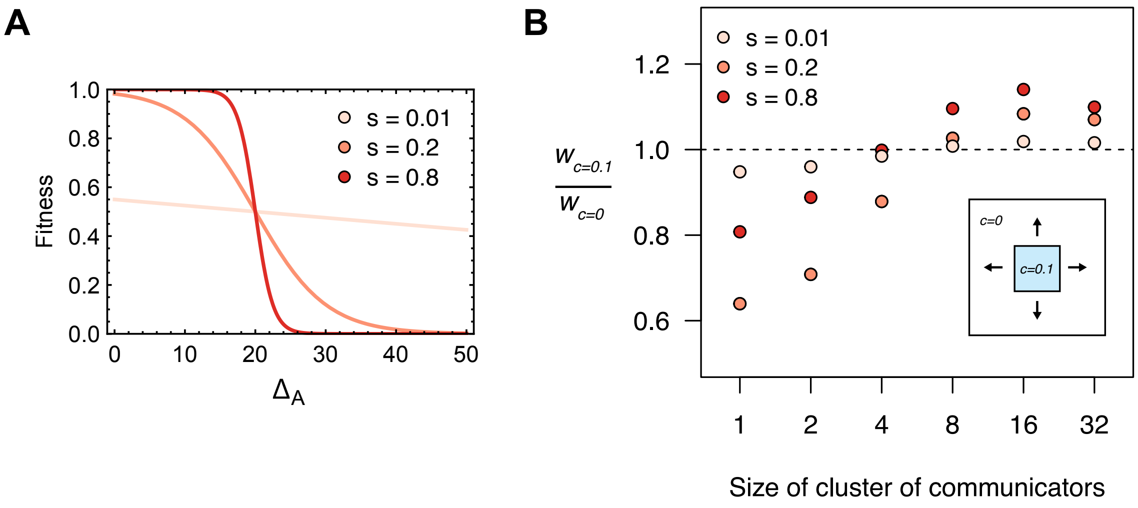

Figure S5: **Steeper fitness functions favor the evolution of collective sensing.** A) Fitness function for varying steepness values. B) Mean fitness of a cluster of communicators ($c = 0.1$) in the center of the 50x50 grid relative to the mean fitness of the remaining population that does not communicate ($c = 0$) for the different fitness functions displayed in A). The size of the cluster of communicators increases along the x-axis (as illustrated by the inset). Fitness values are calculated by simulating two environmental cycles –one in the 'ON' and one in the 'OFF' state– and averaging over all the cells with the same value of c. Each dot corresponds to the average of the ratio between the mean fitness values of cells with $c = 0.1$ and $c = 0$ over 200 replicate simulations. For very low values of s ($s = 0.01$) the fitness function becomes almost flat so there is a small incentive for cells to correctly determine the state of the environment and thus for collective sensing to evolve. For intermediate values of s ($s = 0.2$) the shape of the fitness function is less sigmoidal so cells are penalized even for small deviations from the optimal value of A. This reduces the advantage of small clusters of communicators which might make the correct estimate of the environment but take longer to synchronize and thus deviate slightly from the optimal value of A when averaging over the entire environmental cycle.

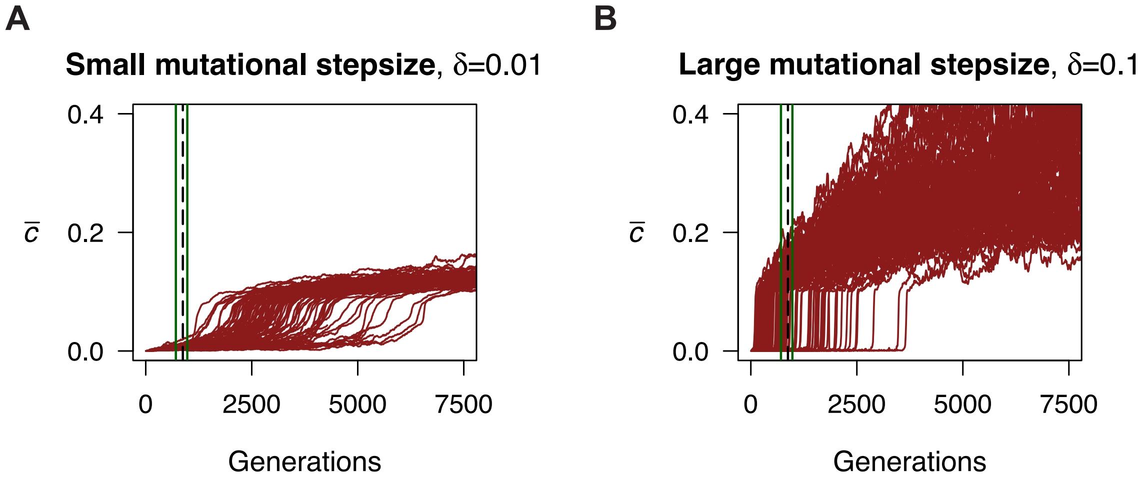

Figure S6: **Intermediate mutational stepsizes favor the evolution of collective sensing.** A) Mean c in 100 replicate evolutionary simulations with the same parameters as in Fig. 2 but a smaller mutational stepsize ($\delta = 0.01$). The mean time for the evolution of communication in Fig. 2 ($\delta = 0.03$) is shown by the dotted line with the first and third quartiles shown by green lines. With a small mutational step it takes considerably longer for a minimum number of communicators with high enough c to emerge relative to an intermediate mutational step (like the one in Fig. 2). This slows down the emergence of collective sensing. B) Mean c in 100 replicate evolutionary simulations with the same parameters as in Fig. 2 but a larger mutational stepsize ($\delta = 0.1$). The solid and dotted lines are as in panel A). With a large mutational stepsize communicators arise often relative to an intermediate stepsize (like the one in Fig. 2). While sometimes this leads to the rapid evolution of collective sensing, these mutants also go extinct frequently because in the absence of other communicators high values of c are detrimental - they turn the dynamical system monostable and sensitive to the extracellular concentration of A which is not informative if other cells are not communicating. For this reason, there is large variance in the time to the evolution of communication when the mutational stepsize is large and in many simulations it takes long for collective sensing to evolve.

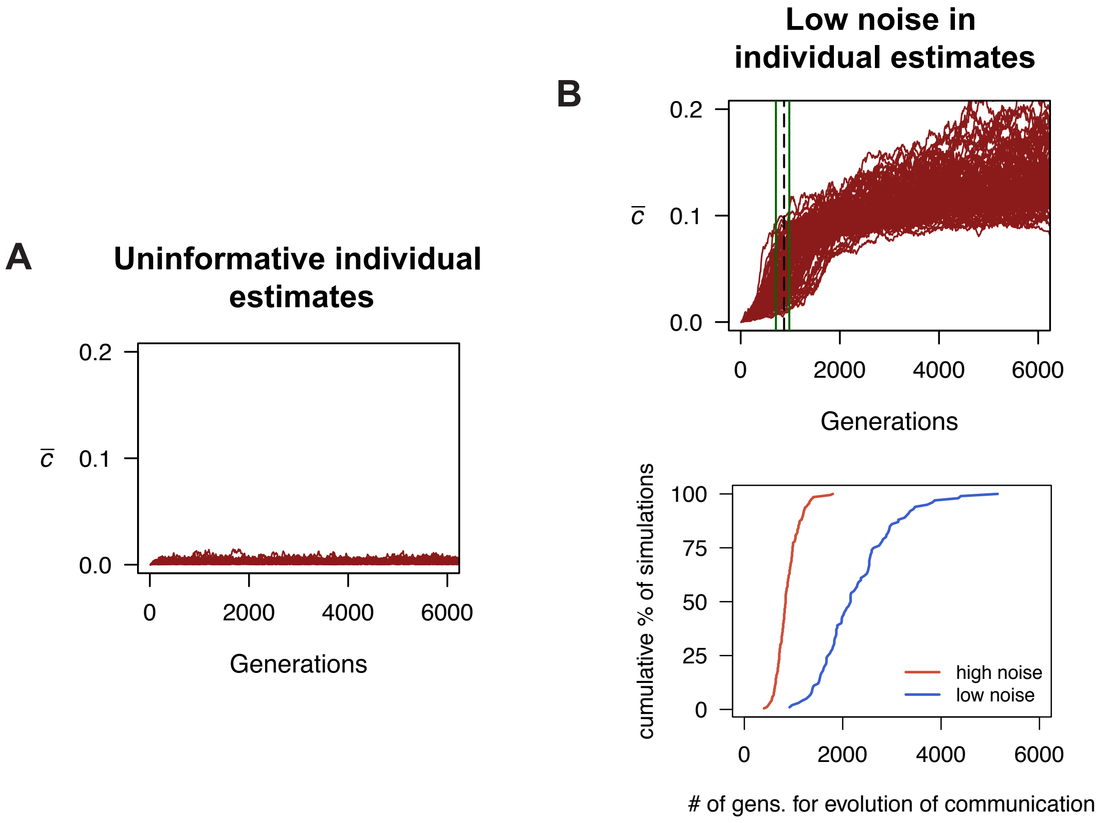

Figure S7: **The evolution of collective sensing depends on the estimation accuracy of individual cells.** A) Mean c in 100 replicate evolutionary simulations where bacteria sample the initial intracellular concentration of A from the same distribution regardless of the state of the environment. Other parameters are the same as in Fig. 2. When bacteria are unable to individually estimate the state of the environment, collective sensing is not profitable and does not evolve. B) (*top*) Mean c in 100 replicate evolutionary simulations with the same parameters as in Fig. 2 but with lower noise in the individual estimates of the environmental conditions ($\sigma_{\mathrm{OFF}} = 10$ and $\sigma_{\mathrm{ON}} = 20$). The mean time for evolution of communication with higher noise in the individual estimates of the environment is shown by the black dotted line (Fig. 2, $\sigma_{\mathrm{OFF}} = 25$ and $\sigma_{\mathrm{ON}} = 50$), with the first and third quartiles indicated by green lines. (*bottom*) Cumulative distributions of the time to fixation of cell-cell communication in the two scenarios compared in the figure above. We define communication as fixed in the population when $\bar{c} > 0.1$ (see Fig. S4). Since the benefit of collective sensing comes from the error that cells make when estimating environmental conditions, lower noise in such estimates makes communication less profitable and collective sensing takes longer to evolve.

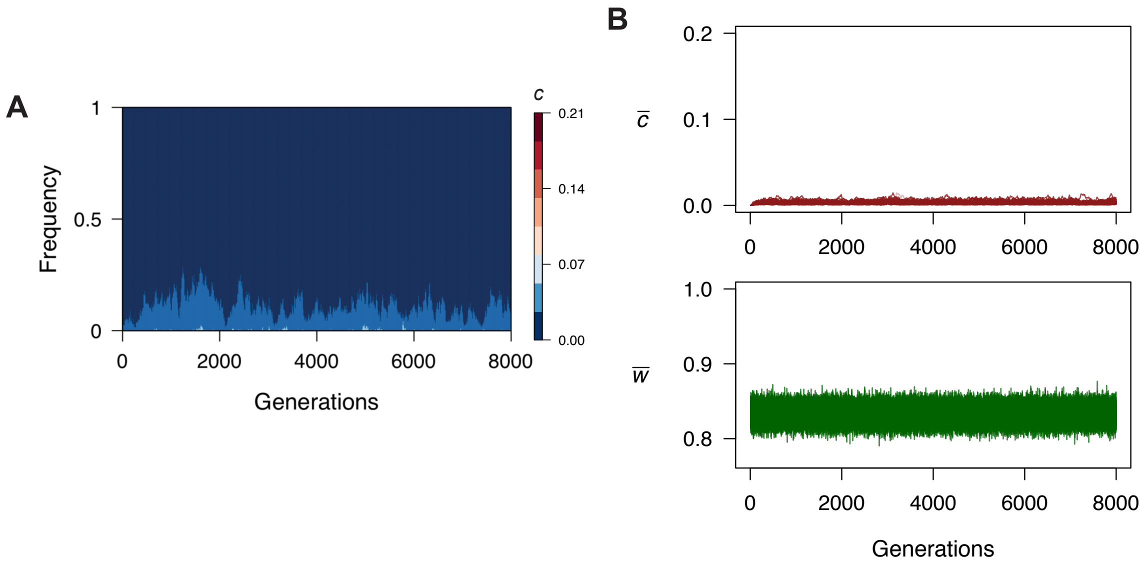

Figure S8: **Communication does not evolve in the absence of extracellular diffusion.** A) Evolution of the communication parameter c across time in a single evolutionary simulation where $D = 0$. B) Mean c (top) and mean population fitness (bottom) across 7500 generations in 50 replicate evolutionary simulations where $D = 0$. Other parameters are the same that in Fig. 2. c does not evolve to high values in the absence of environmental diffusion. Thus, cells only benefit from exchanging A with the extracellular environment because they can communicate with other cells and not because they can gather information about the current environment from the initial concentration of A in the extracellular space.

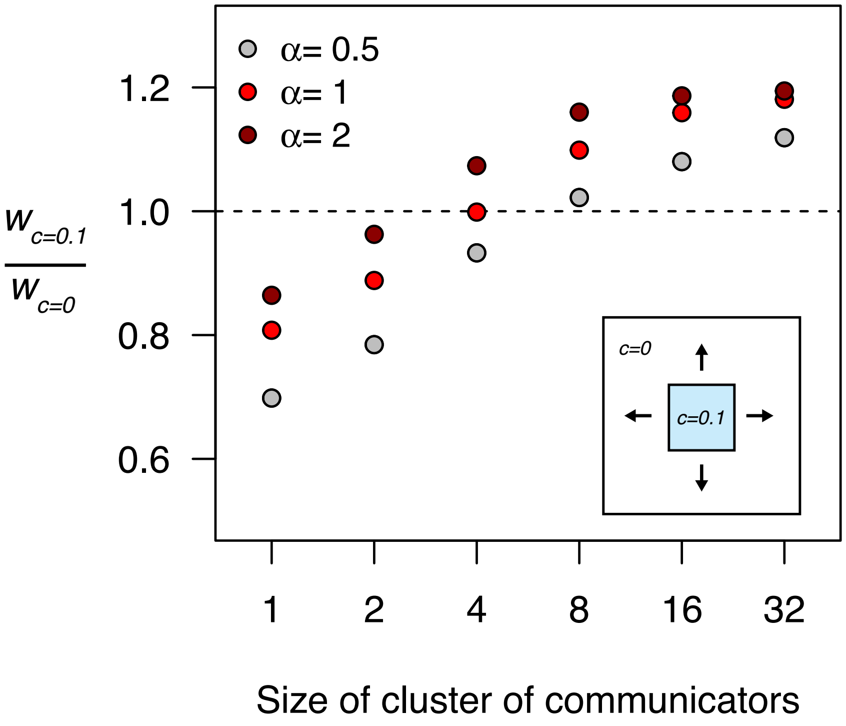

Figure S9: **Large extracellular volumes reduce the efficiency of collective sensing.** Mean fitness of a cluster of communicators ($c = 0.1$) in the center of the 50x50 grid relative to the mean fitness of the rest of the population that does not communicate ($c = 0$). The size of the cluster increases along the x-axis (as illustrated by the inset) and the colors correspond to different values of the rescaling parameter α (with lower values of α corresponding to higher extracellular volumes). Fitness values are calculated by simulating two environmental cycles -one in the 'ON' and one in the 'OFF' state- and averaging over all the cells with the same value of c. Each dot corresponds to the average of the ratio between the mean fitness values of cells with $c = 0.1$ and $c = 0$ over 200 replicate simulations. Since the extracellular concentration of A is initially set to a random value between 0 and $A_{\text{OFF}}+A_{\text{ON}}$ in each grid cell, this concentration is uninformative to bacteria unless they can actively modify it. When the total extracellular volume increases bacteria have less influence over the extracellular concentration of A and as a result, collective sensing becomes less efficient (i.e. larger clusters of communicating cells are needed for communication to be profitable).

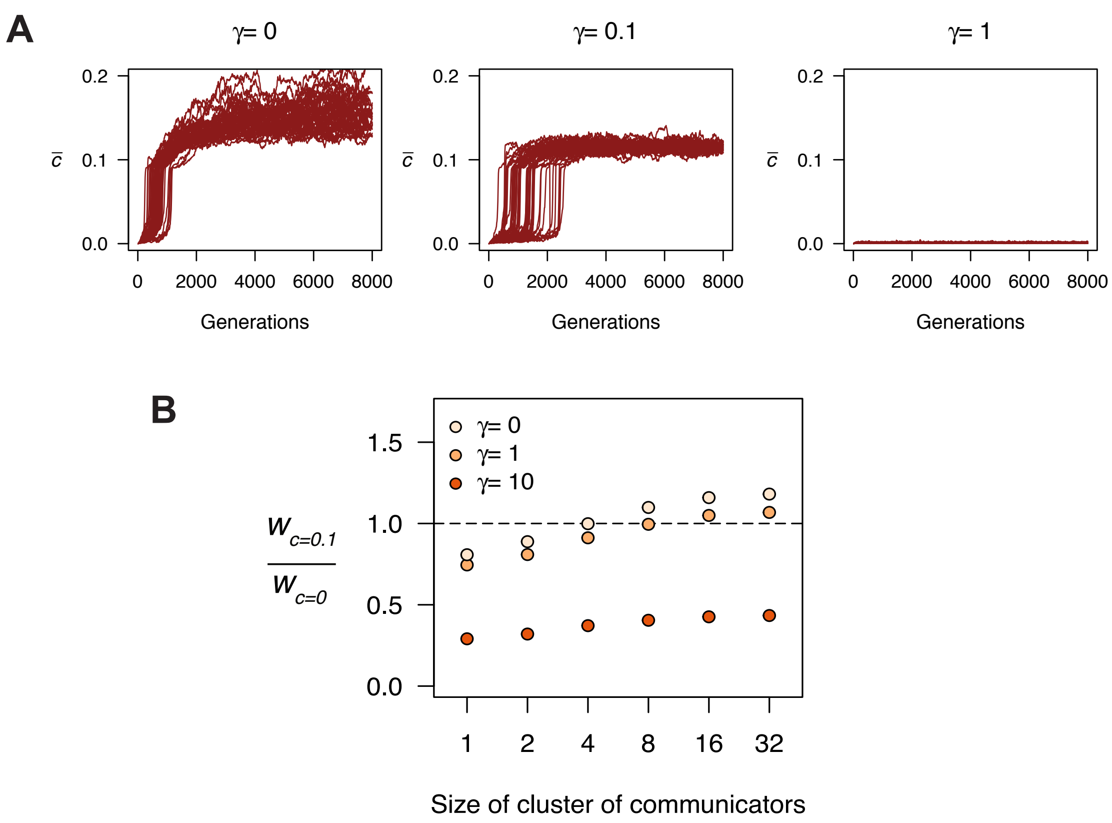

Figure S10: **The evolution of collective sensing is impeded by very high costs of communication**. A) Mean c across 8000 generations in 100 replicate evolutionary simulations with increasing cost of communication γ. B) Mean fitness of a cluster of communicators ($c = 0.1$) in the center of the 50x50 grid relative to the mean fitness of the remaining population that does not communicate ($c = 0$). The size of the cluster increases along the x-axis and the colors correspond to different values of γ. Fitness values are calculated by simulating two environmental cycles -one in the 'ON' and one in the 'OFF' state- and averaging over all the cells with the same value of c. Each dot corresponds to the average of the ratio between the mean fitness values of cells with $c = 0.1$ and $c = 0$ over 200 replicate simulations. Costs of communication reduce the advantage of collective sensing and can impede its evolution when they become too large.

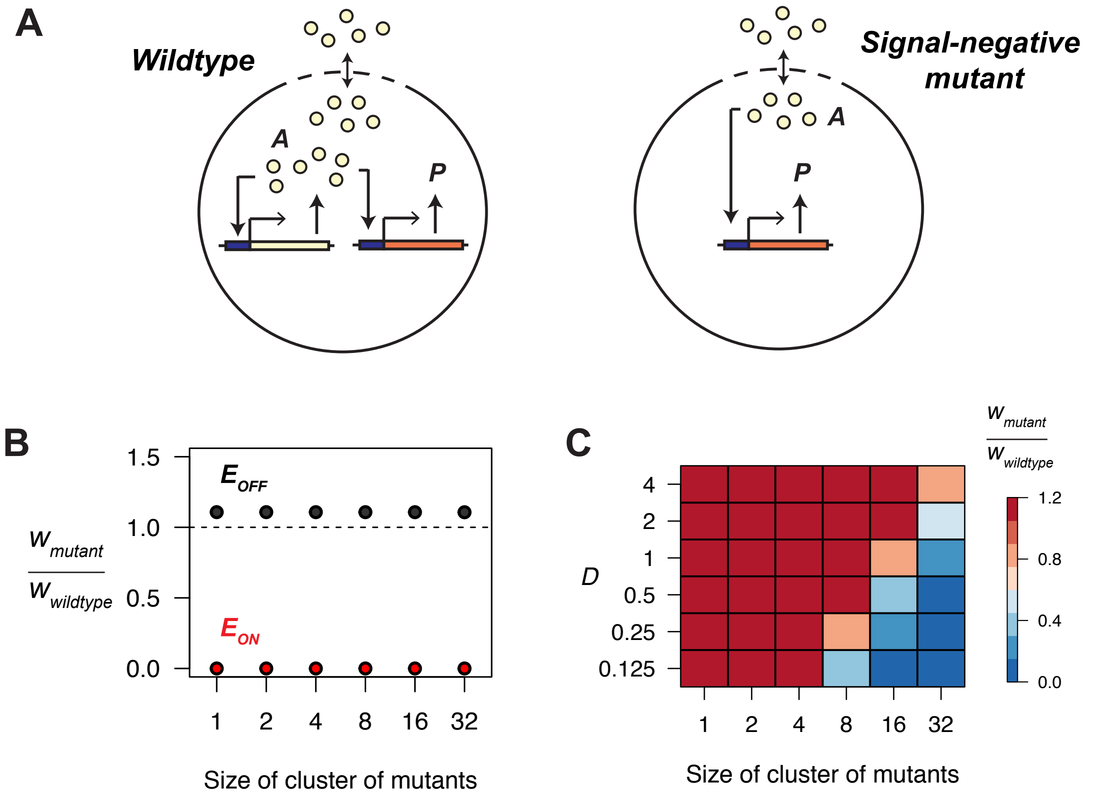

Figure S11: **Signal-negative mutants are subject to negative frequency-dependent selection**. A) Model extension to study the evolution of signal-negative mutants. Fitness depends on the production of a product P that is under the control of A. Both wildtype and signal-negative mutants exchange A with the extracellular environment. However, wildtype cells produce A, while signal-negative mutants save the cost of production and rely on importing A secreted by other cells. B) Mean fitness of a cluster of signal-negative mutants in the center of the 50x50 grid, relative to the mean fitness of the remaining population (consisting of wildtype cells) in the two environmental states. The size of the cluster increases along the x-axis. We assume that both cell types have $c = 0.1$ and that the fitness of wildtype cells is reduced by 10% due to A production. Signal-negative mutants have an advantage in E_{OFF} environments, while the opposite occurs in E_{ON} environments where they cannot produce enough P since they rely on A produced by other cells. Mutants can resolve this deficiency by becoming more sensitive to A (panel C). C) Mean fitness of a cluster of signal-negative mutants that are highly sensitive to A, relative to the mean fitness of the remaining population in an E_{ON} environment as a function of the size of the mutant population and the rate of environmental diffusivity, D. Because these mutants avoid the cost of communication and are highly sensitive to A, they can outperform the wildtype when they are in low frequency even in an E_{ON} environment. However, these mutants succumb to their own success when they increase in number, because they erode the benefit of collective sensing in their local environment. Since these mutants rely on AIs produced by the wildtype, their fitness also decreases with the diffusivity of the environment, D. Each data point in B) and C) corresponds to an average over 200 replicate simulations. To model the increased sensitivity of the mutants to A, we assume that their $K = 5$ as opposed to $K = 50$ for the wildtype.
